# “Association of blood cell indices and anemia with risk of incident dementia”: Missing important covariates in MRI analysis may be misleading

**DOI:** 10.1002/alz.13547

**Published:** 2023-11-27

**Authors:** Xinjie Zhang, Wenwu Zhou

**Affiliations:** ^1^ Department of Pediatric Neurosurgery, West China Second University Hospital Sichuan University Chengdu Sichuan China; ^2^ Key Laboratory of Birth Defects and Related Diseases of Women and Children (Sichuan University) Ministry of Education Chengdu Sichuan China

This letter is in response to “Associations of blood cell indices and anemia with risk of incident dementia: A prospective cohort study of 313,448 participants” by Qiang and colleagues.^1^

Brain magnetic resonance imaging (MRI) is an important tool for understanding brain structure, offering an opportunity to evaluate the possible mechanisms underlying dementia progression.^2^, ^3^ In their recent article, Qiang and colleagues assessed associations of blood cell indices and anemia with the risk of dementia and brain MRI measures. However, two crucial questions were not well addressed in the MRI data analysis. First, the presence of chronic brain disorders (including stroke, Parkinson's disease, cerebral hemorrhage, etc.) could significantly affect brain structure and function,^4^, ^5^ and the authors did not adequately account for chronic brain disorders in their analysis. Simply adjusting for the history of stroke at baseline was insufficient, and given that the MRI of UKB was measured about 9 years after baseline, the estimated associations of blood cell indices and anemia with MRI measures should be more accurate if adjusted for or excluded for chronic brain disorders (ie, before MRI assessment). Second, according to the official UKB MRI documentation, the exact location of the head and the radio‐frequency receive coil in the scanner can (including the X‐position of center‐of‐gravity of brain mask in scanner co‐ordinates, Y‐position of back of brain mask in scanner co‐ordinates, Z‐position of center‐of‐gravity of brain mask in scanner co‐ordinates, and Z‐position of table/coil in scanner co‐ordinates) affect data quality and imaging‐derived phenotypes and can be used as “confounding variables.”^6^, ^7^ To the best of our knowledge, controlling these confounds may have a large impact on the estimations or even reverse the results, as they are directly relevant to the quality of the MRI data.

If the authors further consider these important confounders, it is believed that their conclusions will be more reliable.

## CONFLICT OF INTEREST STATEMENT

The authors declare no conflicts of interest. Author disclosures are available in the supporting information.

## Supporting information

Supporting Information
